# Nuclear DNA markers for identification of Beluga and Sterlet sturgeons and their interspecific Bester hybrid

**DOI:** 10.1038/s41598-017-01768-3

**Published:** 2017-05-10

**Authors:** Miloš Havelka, Takafumi Fujimoto, Seishi Hagihara, Shinji Adachi, Katsutoshi Arai

**Affiliations:** 1 Hokkaido University, Faculty and Graduate School of Fisheries Sciences, 3-1-1 Minato, Hakodate, Hokkaido 041-8611 Japan; 2 University of South Bohemia in Ceske Budejovice, Faculty of Fisheries and Protection of Waters, South Bohemian Research Center of Aquaculture and Biodiversity of Hydrocenoses, Research Institute of Fish Culture and Hydrobiology, Zátiší 728/II, 389 25 Vodňany, Czech Republic

## Abstract

Sturgeons (Acipenseriformes) are among the most endangered species in the world due to fragmentation and destruction of their natural habitats and to overexploitation, mainly for highly priced caviar. This has led to the development of sturgeon culture, originally for reintroduction, but more recently for caviar production. In both cases, accurate species identification is essential. We report a new tool for accurate identification of *Huso huso* and *Acipenser ruthenus* based on nuclear DNA markers. We employed ddRAD sequencing to identify species-specific nucleotide variants, which served as specific binding sites for diagnostic primers. The primers allowed identification of *Huso huso* and *Acipenser ruthenus* as well as their discrimination from *A*. *baerii*, *A*. *schrenckii*, *A*. *gueldenstaedtii*, *A*. *stellatus*, *A*. *persicus*, *A*. *mikadoi*, *A*. *transmontanus*, *and H*. *dauricus* and identification of *A*. *ruthenus and H*. *huso* hybrids with these species, except hybrid between *A*. *ruthenus* and *A*. *stellatus*. The species-specific primers also allowed identification of bester (*H*. *huso* × *A*. *ruthenus*), the most commercially exploited sturgeon hybrid. The tool, based on simple PCR and gel electrophoresis, is rapid, inexpensive, and reproducible. It will contribute to conservation of remaining wild populations of *A*. *ruthenus* and *H*. *huso*, as well as to traceability of their products.

## Introduction

Sturgeon (Acipenseriformes) are ancient fish with an evolutionary history of more than 200 million years^1^. Natural populations have declined during the past century through poaching for caviar, water pollution, and habitat degradation, making them currently the world’s most endangered group of species. Seventeen species are classified as critically endangered^2^, with most populations continuing to decrease, and the extinction of some is highly probable^3^.

Decline in catches over the past 50 years has led to the development of sturgeon culture, originally for reintroduction, but more recently for caviar production^4^. To meet the market demand for sturgeon products, dedicated aquaculture techniques have been developed, and sturgeon hybrids have become widely implemented^5^. Similar to other fish hybrids, sturgeon hybrids are reared mainly for better performance compared to parent species (hybrid vigour). Besides production of meat, full fertility of cultured sturgeon hybrids allows their use in caviar production.

Accurate species identification is a necessary prerequisite of any reintroduction program and also has importance for regulating trade of high value animal products. High morphological plasticity^6^ and frequent interspecific hybridization^7^ preclude identification of sturgeon species based on morphology, as is commonly used in ichthyology. Molecular markers can overcome this problem. Proposed markers for identification of sturgeon are mitochondrial DNA (mtDNA), random amplified polymorphic DNA (RAPD), and amplified fragment length polymorphic (AFLP) DNA markers^8^. However, mtDNA has limitations for identification of hybrids due to maternal inheritance, and RAPD and AFLP have low reproducibility and are costly and time consuming. Five species of sturgeon, *Acipenser naccarii*, *Acipenser fulvescens*, *Acipenser stellatus*, *Acipenser sinensis*, *and Acipenser transmontanus* and their hybrids can be unambiguously identified by nuclear DNA markers^9^. Recently, Boscari, *et al*.^10^ developed nuclear marker allowing identification of a specimen having *H*. *huso* as a parental species.

The goal of this study was to develop a molecular tool for routine identification of *Huso huso*, and *Acipenser ruthenus* as well as their hybrid, the bester. *Huso huso* is among the most endangered of Acipenseriformes, and its caviar is the most costly in the trade. Due to a shortage of wild populations, interest in farming *H*. *huso* for caviar production has grown. Less valuable roe from other species or hybrids is sometimes fraudulently sold as *H*. *huso* caviar^11^. *Acipenser ruthenus* is an ecologically valuable species in the Danube drainage, where it is endangered at population level. Various hybrids of both species have been reported in nature^11–13^. This may contribute to a decline in their populations and disrupt reintroduction programs. *Huso huso* females and *A*. *ruthenus* males are used for the production of the bester, one of the most frequent commercially exploited sturgeon hybrids. Bester products are easily interchangeable with *H*. *huso* products and impossible to discriminate by mtDNA. A reliable tool for unambiguous identification of pure *H*. *huso* and *A*. *ruthenus* and the bester hybrid is highly desirable and may significantly contribute to conservation efforts for both species as well as to global trade control of their products.

We used double-digest restriction-associated DNA (ddRAD) sequencing, which allowed identification of species-specific nucleotide variants to be used for design of diagnostic primers. The primers ensured identification of *H*. *huso*, *A*. *ruthenus*, and bester, as well as their discrimination from eight other species: *Acipenser baerii*, *Acipenser schrenckii*, *Acipenser gueldenstaedtii*, *A*. *stellatus*, *Acipenser persicus*, *Acipenser mikadoi*, *A*. *transmontanus*, *and Huso dauricus*. The tool, based on simple PCR and gel electrophoresis, is rapid, inexpensive, and reproducible. It also allows identification of hybrids of *A*. *ruthenus and H*. *huso* with the mentioned species, except hybrid between *A*. *ruthenus* and *A*. *stellatus*, without requiring a specific marker for the species with which *A*. *ruthenus* or *H*. *huso* is crossed. Excluding hybrids from sturgeon breeding programs is essential, as hybridization is considered the most rapidly acting genetic threat to endangered populations^14^. In the trade, lower priced caviar from hybrids should be detected to avoid mislabeling and to protect highly valued single-species caviar.

## Results

### Identification of *A*. *ruthenus*

**Figure 1:**
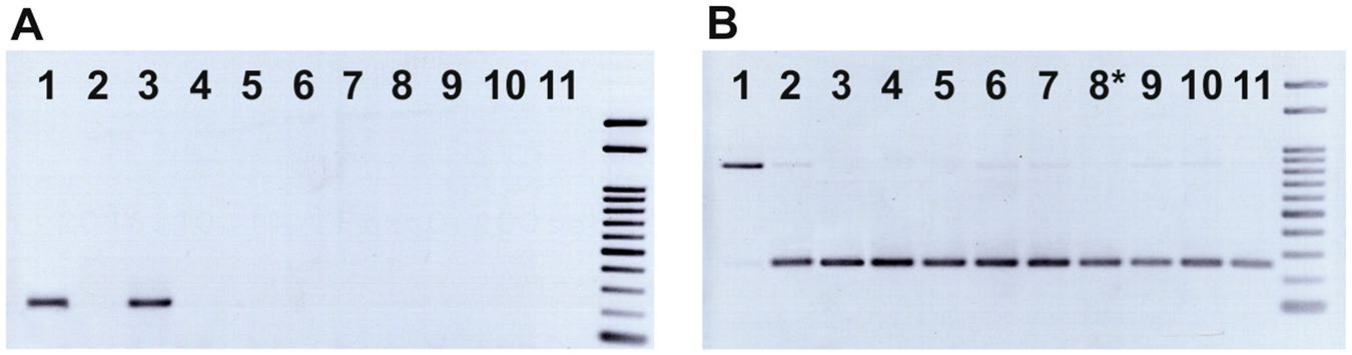
Expected amplification given by *A*. *ruthenus* positive primer 247_ARp (**A**) and *A*. *ruthenus* negative primer 247_ARn (**B**) in combination with common primer 247_uni. Amplification of a 750 bp band was occasionally provided by *A*. *ruthenus* negative primer 247_ARn, but this amplification was not species-specific. 1 = *A*. *ruthenus*; 2 = *H*. *huso*; 3 = bester; 4 = *A*. *baerii*; 5 = *H*. *dauricus*; 6 = *A*. *schrenckii*; 7 = *A*. *gueldenstaedtii*; 8 = *A*. *stellatus*; 9 = *A*. *persicus*; 10 = *A*. *mikadoi*; 11 = *A*. *transmontanus*. *Positive amplification was obtained only in 57,5% samples of *A*. *stellatus*.

We found one dinucleotide variant represented by AG nucleotide bases in reference contig n. 140238 and in all 36 reads of *A*. *ruthenus* aligned to that contig, while all 39 reads of *H*. *huso* and all 78 reads of *A*. *baerii* aligned to that contig had CT nucleotide bases at the same position. This variant was considered diagnostic for *A*. *ruthenus* and was used for design of the *A*. *ruthenus* primers (Table 1). The dinucleotide variant (AG) in *A*. *ruthenus* reads determined the binding of *A*. *ruthenus* positive primer 247_ARp (Supplementary information). The dinucleotide variant (CT) in reads of *A*. *baerii* and *H*. *huso* determined the binding of *A*. *ruthenus* negative primer 247_ARn (Supplementary information). Using *A*. *ruthenus* positive primer 247_ARp with common primers 247_uni, we obtained 100% amplification of a 247 bp fragment in 120 *A*. *ruthenus* samples, with no amplification in any specimen of other analyzed species (Fig. 1A). On the contrary, no amplification in 120 *A*. *ruthenus*, but 100% amplification of a 247 bp fragment in all specimens of other analyzed species except *A*. *stellatus*, was observed when using *A*. *ruthenus* negative primer 247_ARn in combination with primer 247_uni (Fig. 1B). In *A*. *stellatus*, 23 samples had positive amplification, while 17 samples showed no amplification when using *A*. *ruthenus* negative primer 247_ARn in combination with primer 247_uni. Amplification of a 750 bp band was occasionally provided by *A*. *ruthenus* negative primer 247_ARn in combination with primer 247_uni, but this amplification was not species-specific (Fig. 1B

**Table 1:** Primers developed for identification of *Huso huso* and *Acipenser ruthenus* and validation tests of all primer pairs performed on nine sturgeon species and bester, hybrid of *H*. *huso* and *A*. *ruthenus*.

Primers			Tested species										
Pairs	Sequence 5′–3′	bp	*Hh*	*Ar*	*Ab*	*Hd*	*Asch*	*Ag*	*Ast*	*Ap*	*Am*	*At*	BE
153_HHp	GATCTGAACATCAGCCACTGC	153	**47/47**	0/120	0/40	0/17	0/18	0/38	0/40	0/21	0/8	0/32	24/24
153_uni	TACTGTGCCTGTATGTCTCC												
153_HHn	GATCTGAACATCAGCCACTGG	153	0/47	120/120	40/40	17/17	18/18	38/38	40/40	21/21	8/8	32/32	24/24
153_uni	TACTGTGCCTGTATGTCTCC												
247_ARp	TAAGGGTCCATGCATGCAG	247	0/47	**120/120**	0/40	0/17	0/18	0/38	0/40	0/21	0/8	0/32	24/24
247_uni	TTTTAGCTGCACCGTGGC												
247_ARn	TAAGGGTCCATGCATGCCT	247	47/47	0/120	40/40	17/17	18/18	38/38	23/40	21/21	8/8	32/32	24/24
247_uni	TTTTAGCTGCACCGTGGC												

).

### Identification of *H*. *huso*

**Figure 2:**
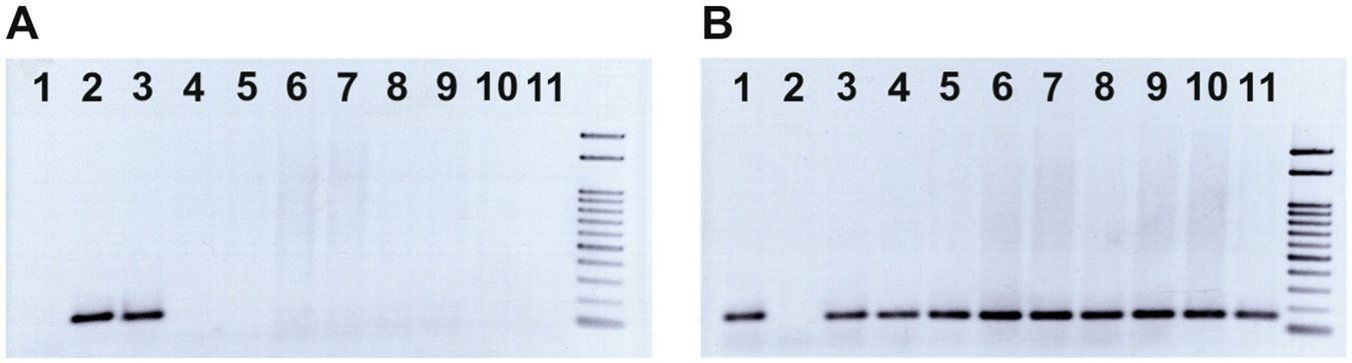
Expected amplification given by *H*. *huso* positive primer 153_HHp (**A**) and *H*. *huso* negative primer 153_HHn (**B**) in combination with common primer 153_uni. 1 = *A*. *ruthenus*; 2 = *H*. *huso*; 3 = bester; 4 = *A*. *baerii*; 5 = *H*. *dauricus*; 6 = *A*. *schrenckii*; 7 = *A*. *gueldenstaedtii*; 8 = *A*. *stellatus*; 9 = *A*. *persicus*; 10 = *A*. *mikadoi*; 11 = *A*. *transmontanus*.

We found six private single nucleotide variants in *H*. *huso*. These were considered as putatively diagnostic and used for primer design. The diagnostic variant that ensured discrimination of *H*. *huso* was represented by nucleotide base C in all 509 reads aligned to corresponding *A*. *ruthenus* reference contig n. 216845. This variant determined the binding of *H*. *huso* positive primer 153_HHp (Supplementary information). The reference contig n. 216845, all 484 aligned reads of *A*. *ruthenus*, and all 740 aligned reads of *A*. *baerii* possessed nucleotide base G at the same position, which determined the binding of *H*. *huso* negative primer 153_HHn (Supplementary information). Using *H*. *huso* positive 153_HHp primer with common primers 153_uni, we obtained 100% amplification of a 153 bp fragment in all 47 *H*. *huso* samples and in a caviar sample, with no amplification in any specimen of other analyzed species (Table 1; Fig. 2A; Supplementary information). On the contrary, 100% amplification of 153 bp fragments in all specimens of other analyzed species and no amplification in 47 *H*. *huso* and the caviar sample were observed when using *H*. *huso* negative primer 153_HHn in combination with primer 153_uni (Table 1; Fig. 2B; Supplementary information).

### Identification of bester

The 204 reads of bester aligned to reference contig n. 216845 contained 95 reads with the *H*. *huso* specific variant (C) and 109 reads with variant (G). The bester reads aligned to reference contig n. 140238 contained 9 reads with the *A*. *ruthenus* specific variant (AG) and 7 reads with variant (CT). *Huso huso* positive primer 153_HHp and *A*. *ruthenus* positive primer 247_ARp ensured amplification of fragments of 153 bp and 247 bp, respectively, in all 24 analyzed bester specimens (Figs 1A and 2A). Both species-negative primers (153_HHn and 247_ARn) provided successful amplification of corresponding fragments (Figs 1B and 2B).

## Discussion

**Figure 3:**
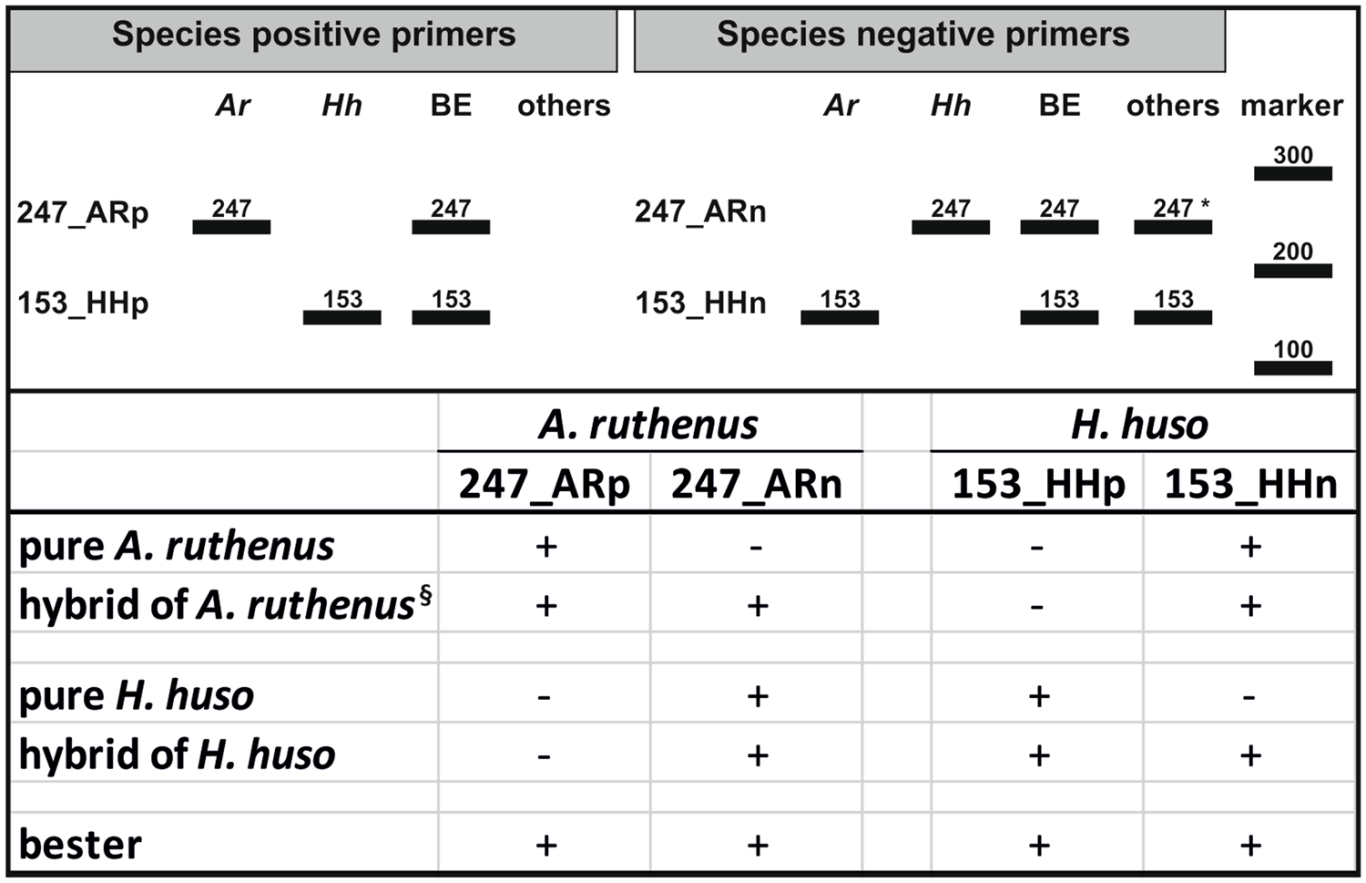
Expected band patterns for identification of *H*. *huso* (*Hh*), *A*. *ruthenus* (*Ar*), bester (BE), and for other tested species (others), using *A*. *ruthenus and H*. *huso* positive and negative primers. + = positive amplification; − = negative amplification. *Positive amplification of 247 bp band was obtained only in 57,5% samples of *A*. *stellatus*; ^§^except hybrid between *A*. *ruthenus* and *A*. *stellatus*.

We present a new tool for identification of *A*. *ruthenus* and *H*. *huso* using simple dominant bi-allelic nuclear DNA markers (presence/absence of a given band) that allow discrimination of *A*. *ruthenus and H*. *huso* from eight other sturgeon species: *A*. *baerii*, *A*. *schrenckii*, *A*. *gueldenstaedtii*, *A*. *stellatus*, *A*. *persicus*, *A*. *mikadoi*, *A*. *transmontanus*, and *H*. *dauricus* (Table 1). The combination of species-positive and species-negative primers also allows detection of hybrids of *A*. *ruthenus and H*. *huso* with the tested species, except hybrid between *A*. *ruthenus* and *A*. *stellatus* (Fig. 3).

Our approach is based on nuclear DNA variants identified by ddRAD sequencing. RAD sequencing has been previously used to reveal molecular genetic markers that differentiate *A*. *gueldenstaedtii* from *A*. *persicus* ^15^. Rather than characterize hundreds of SNPs, we focused on identifying homozygous variants private to given species that determine specific binding for diagnostic primers, and on the design and validation of such primers.

Identification of target species consisted of two steps, PCR reaction with the species-positive primer to determine presence or absence of the species genome in the tested sample and PCR using the species-negative primer. Amplification by the positive primer, but no amplification with the negative primer, identified the sample as pure *H*. *huso* or *A*. *ruthenus* (Fig. 3). Amplification by both positive and negative primers indicated a hybrid of *H*. *huso* or *A*. *ruthenus* with one or more of the tested species (Fig. 3). The exception from this pattern was observed in *A*. *stellatus*. As expected, the *A*. *ruthenus* positive primer had no amplification in all *A*. *stellatus* samples. However, the *A*. *ruthenus* negative primer ensured amplification only in 27 for 40 samples of *A*. *stellatus*. Thus, our tool allowed 100% discrimination between *A*. *stellatus* and *A*. *ruthenus* genome, but capability of detecting hybrid of these two species was only 57.5%. For unambiguous identification of hybrid between *A*. *ruthenus* and *A*. *stellatus*, we recommend using combination of our tool and *A*. *stellatus* specific primer developed by Boscari, *et al*.^9^.

In a previous study, a 10 bp deletion in the first intron of ribosomal protein S7 allowed discrimination of *A*. *ruthenus* and *A*. *baerii* from other sturgeon species with efficacy of 96% and 60.6%, respectively^9^. However, it did not discriminate between these two species, and there was no nucleotide variability in that intron allowing discrimination of *H*. *huso* ^9^. Recently, Boscari, *et al*.^10^ developed a tool for identification of *H*. *huso* based on the species-specific SNP at the second intron of the S6 Ribosomal Protein. Contrary to our approach, the tool proposed by Boscari, *et al*.^10^ does not allow discrimination between pure *H*. *huso* and its hybrids without requiring specific markers for other sturgeon species with which *H*. *huso* might be crossed.

Natural populations of *H*. *huso* have been dramatically reduced due to poaching and habitat degradation^16^, and survival of the species is highly dependent on artificial breeding programs. Many populations of *A*. *ruthenus* species are also undergoing serious decline, especially in the upper and middle Danube^17^, and restocking is planned or already in progress^18^. Hybrids of *H*. *huso* with *A*. *stellatus*, *A*. *gueldenstaedtii*, and *Acipenser nudivetris* have been reported in nature^11, 12^. Hybrids of *A*. *ruthenus* with *A*. *baerii* have been observed in the Danube River^13^. These hybrids may originate from natural hybridization, reintroduction or escapees from aquaculture. In any case, our tool may contribute to identification of pure *A*. *ruthenus* and *H*. *huso* and prevent the undesirable presence of their hybrids among broodstock. This is essential for conservation and reintroduction.

*Huso huso* provides high-value caviar that is occasionally substituted with a less desirable product from other sturgeon species or hybrids^19^. Unambiguous identification of *H*. *huso* roe to distinguish it from roe of other species and hybrids is the only way to prevent mislabeling and commercial fraud. This is especially important for *H*. *huso* hybrids, including bester. Our tool works for caviar samples and requires only one roe for analysis. Thus, it may be used for routine identification of *H*. *huso* caviar.

*Acipenser ruthenus* is used as a model species in sturgeon research due to ease of handling, short maturation time, and its routine reproduction in captivity^20, 21^. The marker for *A*. *ruthenus* discrimination is easily applicable to primary research in sturgeon.

Due to incredible rarity of *A*. *mikadoi*, we had only 8 samples available for primer validation. *Acipenser mikadoi* is from different clade than *A*. *ruthenus* and *H*. *huso* ^22^. Therefore, it is unlikely that *A*. *mikadoi* shares diagnostic variant with *A*. *ruthenus* and *H*. *huso*, if no other species in the Pacific clade possess it, and the likelihood of a random mutation at that exact base is negligible.

Identification of bester is based on a simple test using *A*. *ruthenus and H*. *huso* positive primers. This can be done in a single reaction mix, as both positive primer pairs have the same annealing temperature. Positive amplification in *H*. *huso* is determined by a 153 bp band, and, in *A*. *ruthenus*, by a 247 bp band. Both bands will be present only if a sample is a hybrid between *A*. *ruthenus and H*. *huso*. The method does not allow discrimination of species on the maternal position. This may be accomplished by additional analysis of mtDNA^23^; however, the reciprocal hybrid (*A*. *ruthenus* female × *H*. *huso* male) is not commonly used in aquaculture^24^.

The bester is one of the most commercially utilized sturgeon hybrid, owing to rapid growth rate and high quality eggs derived from the maternal *H*. *huso* along with early maturity and superior flesh quality from paternal *A*. *ruthenus* ^24^. The presence of bester caviar and meat on the market demands a means of accurate identification to avoid mislabeling or their substitution for more costly products from purebred sturgeon.

Accurate species identification should become standard in sturgeon culture. The genetic makeup of all fish should be unambiguously determined prior to their use as broodstock. Sturgeon hybrids might be inadvertently introduced into pure captive broodstock^25, 26^. Thus, currently used broodstock should be also screened. Information on the genetic makeup of a specimen should be accessible and trackable with the specimen and its products. Only this can prevent the undesirable inclusion of hybrids in captive fish bred *ex situ* for conservation and/or caviar production, and commercial frauds. Our tool offers a simple, easily implemented, method of screening specimens and products of *A*. *ruthenus* and *H*. *huso*.

In general, techniques for identification of species and hybrids based on single species-specific nuclear markers allow identification of pure specimens and their F1 hybrids. Efficacy in detecting subsequent hybrid generations (F2, F3,…) and backcrosses decreases following the Mendelian inheritance model of diagnostic variants. In sturgeon aquaculture, F1 hybrids are of greatest interest^27^. The F2 and F3 hybrids and backcrosses are not commonly used, due to diminishing performance, but their occasional occurrence cannot be excluded with absolute certainty. F2 and F3 hybrids and backcrosses can be unambiguously detected only by increasing the number of unlinked diagnostic nuclear markers. Our tool may be combined with other available tools for sturgeon species identification^9, 10^ to expand capability of detecting F2 and F3 hybrids and backcrosses. We recommend this, especially for screening fish captured from the wild and intended for breeding, as backcrosses and F2 hybrids have been reported in wild populations^12^.

## Conclusion

Identification of *A*. *ruthenus and H*. *huso* should become easier with the development of this molecular tool. Since it is based on a simple method using dominant bi-allelic nuclear DNA markers, the protocol is straightforward and thus can be easily implemented across laboratories. Importantly, the markers allow detection of hybrids of *A*. *ruthenus* and *H*. *huso* with any of eight tested species, except hybrid between *A*. *ruthenus* and *A*. *stellatus*, as well as accurate identification of bester, the hybrid that is most commercially exploited.

This technique should contribute to better, more reliable, regulation and control of global trade of high value sturgeon products as well as to their management and conservation.

## Materials and Methods

### Ethics

This study was performed according to the Guide for the Care and Use of Laboratory Animals in Hokkaido University. All animal experiments underwent ethical review and were approved by the Hokkaido University animal care committee (Approval number 19-2). Fish were maintained according to the principles of animal welfare and principles of laboratory animal care based on the Guidelines for the Use of Animals in Research published in Animal Behaviour (1998), 55, 251–257.

### Sampling and DNA extraction

Specimens of *A. ruthenus*, *A. baerii*, *A. schrenckii*, *A. gueldenstaedtii*, *A. stellatus*, *A. persicus*, *A. mikadoi*, *A. transmontanus*, *H. huso*, *H. dauricus*, and bester were collected (Supplementary information). Sixteen samples each from *A. ruthenus*, *A. baerii*, *H. huso*, and bester were processed for ddRAD sequencing. When possible, specimens were selected from different geographic locations. Additionally, a sample of *H. huso* caviar, purchased from a retail outlet in Japan, was included for primer validation (Supplementary information). Genomic DNA was extracted from fin-clips stored in molecular grade ethanol using NucleoSpin® Tissue Kit (MACHEREY-NAGEL, Germany).

### Library preparation and ddRAD sequencing

Library preparation and ddRAD sequencing were performed by IGA Technology Services, Italy. Genomic DNA (200 ng) was incubated with 2U of *SphI-HF* enzyme for 1 h at 37 °C with CutSmart (New England Biolabs) buffer in a reaction volume of 30 uL, followed by heat-inactivation at 65 °C for 20 min. Three units of *BstYI* enzyme was added to the reaction mix and incubated at 60 °C for 1 h. Reaction was inactivated at 65 °C for 20 min. Fragmented DNA was purified with 1.5× volume AMpureXP beads (Agencourt), followed by two 80% ethanol washes and final elution in 20 uL elution buffer (Tris 10 mM – pH 7.5).

Fragments were ligated to barcoded adapters as described in Peterson, *et al*.^28^ and pooled in batches of 24 samples. Size selection was carried out for each pool on 1% low-melting agarose gel, and fragments in the range of 340–490 bp were excised (considering some 80 extra base pairs included by adapter ligation) and purified with QIAquick gel extraction (Qiagen) following manufacturer instructions. Following elution, fragments were PCR enriched with oligos carrying TruSeq indexing sequences as in Peterson, *et al*.^28^ with minor modification: 95 °C for 3 min; 8 cycles of 95 °C for 30 sec, 60 °C for 30 sec, and 72 °C for 45 sec; and 72 °C for 2 min. PCR products were purified with AmpureXP beads as described and sequenced on a HiSeq2500 instrument with V4 chemistry (Illumina) with paired ends of 125 bp each.

### Identification of diagnostic variants

Reads were de-multiplexed for each sample, defined as when reads carried non-ambiguous barcodes (up to 1 mismatch), and restriction sites were consistent on both sides of the fragments (reads 1 and 2). All reads containing uncalled nucleotides were removed from the dataset. Along with the removal of barcode sequences, reads were clipped to a fixed length of 110 bp to remove low-quality bases at the 3′-ends.

Clipped ddRAD-seq reads of *A*. *ruthenus* were initially mapped to *A*. *ruthenus* genome sequence (unpublished data). The draft genome was obtained by Illumina HiSeq2000 sequencing (Macrogen Europe Inc.) from an *A*. *ruthenus* female. The specimen was not involved in ddRAD sequencing, but was used for validation of primers. The *de novo* genome assembly was performed using de Bruijn graphs (K-mar size 23 and bubble size 50) in CLC Genomic Workbench 9.0. The mapping was conducted using CLC Read Mapper implemented in CLC Genomic Workbench 9.0, to improve quality of the genome sequence contigs prior to calling of diagnostic variants. The cost of a mismatch between the read and the reference sequence was set at up to 2, while allowing one gap of length 3. The distance between pair-end reads was detected by the software (CLC Genomics Workbench User Manual v. 9 pages 598–599; http://resources.qiagenbioinformatics.com/manuals/clcgenomicsworkbench/current/User_Manual.pdf). The reference contigs, in which no reads were mapped, were removed. When reads mapped to a contig reference, but there were mismatches to that contig, the contig sequence was updated to reflect the majority base among the reads mapped at that location.

**Figure 4:**
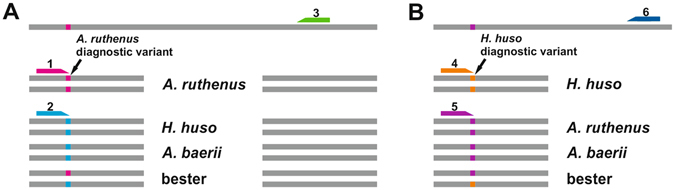
Scheme of read alignment to reference contig of *Acipenser ruthenus*, diagnostic variants, and designed primers. 1 = *A*. *ruthenus* positive primer; 2 = *A*. *ruthenus* negative primer; 3 = common primer; 4 = *H*. *huso* positive primer; 5 = *H*. *huso* negative primer; 6 = common primer.

Calling of putative diagnostic variants was performed by aligning the sequence reads of each species to updated *A*. *ruthenus* genome contigs. The variant that was present in the reference contig and all aligned reads of *A*. *ruthenus*, but in no read of *A*. *baerii* or *H*. *huso*, was defined as *A*. *ruthenus* diagnostic (Fig. 4A). Similarly, the variant that was present in all aligned reads of *H*. *huso*, but not in the reference contig or any read of *A*. *baerii* or *A*. *ruthenus*, was designated *H*. *huso* diagnostic (Fig. 4B). *Acipenser baerii* was included as a non-target species to increase the informative capability of putative diagnostic variants prior to their validation. The variant calling was performed by Variant Detection Tool in CLC Genomic Workbench 9.0. Minimum read coverage for variant calling was set at 32. Finally, putative diagnostic variants of both species were screened for presence in sequencing reads of bester, assuming that both *A*. *ruthenus and H*. *huso* putative diagnostic variants would be present in sequencing reads of their hybrid.

### Primer design and validation

The diagnostic variants were used for primer design, when nucleotides on the 3′ end of the primer were complementary to the diagnostic variants. Preferably, G and C base variants were used to promote specific binding at the 3′ end due to their stronger binding. The di-nucleotide variant was detected and used for primer design in *A*. *ruthenus*. No such di-nucleotide variant was observed in *H*. *huso*; thus, several primers, based on single nucleotide variants, were designed.

To establish a mismatch of two nucleotides if paired with nontarget sequences, the penultimate nucleotide on the 3′ end of the primer was modified to be non-complementary to its target nucleotide. The mismatch of two nucleotides intensifies the failure of amplification when the positive primer is used with non-target sequences. When possible, a reverse primer from each pair was designed to bind a conserved region of given fragment, when any variants in aligned sequencing reads of any species were detected (Fig. 4A). If the reverse primer from each pair could not be designed at that region, it was designed further on the reference contig (Fig. 4B). Using this approach, primer trios were designed for each selected fragment including i) forward primer binding to the diagnostic variant of target species (amplification only in target species), ii) forward primer with no binding to diagnostic variant of target species (amplification only in other analyzed species), and iii) common reverse primers. All primers were designed by Primer 3^29^ implemented in Geneious 6^30^.

The primers were initially tested for amplification in individuals used for ddRAD sequencing by standard gradient PCR. All reactions were performed in a total volume of 25 μL containing 0.25 μM of each primer, 75 mM Tris‐HCl, pH 8.8, 20 mM (NH_4_)_2_SO_4_, 0.01% Tween 20, 2.5 mM MgCl_2_, 800 µM dNTP, 2.5 U Taq‐Purple DNA polymerase, and 25 ng of DNA template. PCR products were inspected on 1.5% agarose gel. Based on this preliminary test, we selected primers that successfully amplified intended fragments, had expected species specificity/non-specificity, and required the same annealing temperature to allow multiplexing. These primers were subsequently tested in 405 specimens of 10 sturgeon species, the bester hybrid, and a sample of commercial caviar (Supplementary information) using the same reaction mix and the following cycling conditions: 95 °C for 120 s; 5 cycles at 95 °C for 60 s, 63 °C for 60 s, and 72 °C for 60 s; 25 cycles at 95 °C for 30 s, 63 °C for 30 s, and 72 °C for 60 s; and a final extension at 72 °C for 12 min. Resulting PCR products were inspected on 1.5% agarose gel.

### Data Accessibility

Alignment of one consensus sequence per species to partial sequence of reference contig 140238 and 216845 are in the Supplementary information.

## Electronic supplementary material

Supplementary Information
