# Supplementary material for: Nuclear DNA markers for identification of Beluga and Sterlet sturgeons and their interspecific Bester hybrid
Source: Sci Rep. 2017 May 10;7:1694. doi: 10.1038/s41598-017-01768-3 (PMC5431886; doi:10.1038/s41598-017-01768-3)
Supplement: Supplementary file 1 — Supplementary Information [file 41598_2017_1768_MOESM1_ESM.pdf]

Supplementary Information

Nuclear DNA markers for identification of Beluga and Sterlet sturgeons and their interspecific Bester hybrid

Miloš Havelka[1,2*], Takafumi Fujimoto[1], Seishi Hagihara[1], Shinji Adachi[1], & Katsutoshi Arai[1]

[1] Hokkaido University, Faculty and Graduate School of Fisheries Sciences, 3-1-1 Minato, Hakodate, Hokkaido 041-8611, Japan

[2] University of South Bohemia in Ceske Budejovice, Faculty of Fisheries and Protection of Waters, South Bohemian Research Center of Aquaculture and Biodiversity of Hydrocenoses, Research Institute of Fish Culture and Hydrobiology, Zátiší 728/II, 389 25 Vodňany, Czech Republic

*Correspondence should be addressed to MH (email: miloshavelka@seznam.cz)

Tel.: +81-(0)138-40-5536

Table S1. Sampling details of all specimens used in the study.

Species	Fish origin	Sampling location+ Samples storage*	Individuals used for ddRAD seq	Individuals used for validation
H. huso	Caspian Sea	FFPW Vodňany+	8	9
	Caspian Sea	Rhönforelle GmbH+	8	18
	Unknown	Rhönforelle GmbH+	–	20
A. ruthenus	Danube River (Hungary)	FFPW Vodňany*	8	24
	Danube River (Slovakia)	FFPW Vodňany*	8	24
	Unknown	FFPW Vodňany+	–	24
	Danube River (Austria)	FFPW Vodňany+	–	24
	Danube River (Serbia and Romania)	FFPW Vodňany*	–	24
Bester	Aquaculture Japan	Hokudai+	16	24
A. baerii	Aquaculture Russia	FFPW Vodňany+	8	21
	Aquaculture Russian, Hungary and Italy	Rhönforelle GmbH+	8	19
H. dauricus	Coast of Hokkaido, Japan	Hokudai*	–	17
A. schrenckii	Aquaculture, Japan	Hokudai*	–	15
	Coast of Hokkaido, Japan	Hokudai+	–	3
A. gueldenstaedtii	Caspian Sea, Aquaculture Russia	FFPW Vodňany+	–	24
	Aquaculture Russia	Rhönforelle GmbH+	–	14
A. stellatus	Azov Sea	FFPW Vodňany+	–	12
	Unknown	Rhönforelle GmbH+	–	28
A. persicus	Unknown	FFPW Vodňany*	–	21
A. mikadoi	Coast of Hokkaido, Coast of Ibaraki, Japan	Hokudai*	–	8
A. transmontanus	Aquaculture Japan	Hokudai*	–	32

cross indicates populations in which live individuals were available for sampling and these individuals are reared in given sampling location

asterisks indicates populations in which fin clips were available for analyses only;

FFPW Vodňany = University of South Bohemia, Faculty of Fisheries and Protection of Waters, Zátiší 728/II, 389 25 Vodňany, Czech Republic

Rhönforelle GmbH = Fischzucht Rhönforelle GmbH & Co. KG, Fischzucht 1, 36129 Gersfeld (Rhön), Germany

Hokudai= Hokkaido University, Faculty and Graduate School of Fisheries Sciences, 3-1-1 Minato, Hakodate, Hokkaido 041-8611, Japan

	5	15	25	35	45	55
	....|....|	....|....|	....|....|	....|....|	....|....|	....|....|
contig_140238_partial_seq	GATCTGGATC	TGAACACTGA	ATTAGGAGTA	TGCTTTTTAA	GACCCTGAGC	TGTAATCAGA
247_AR_*A.ruthenus*_consensus	GATCTGGATC	TGAACACTGA	ATTAGGAGTA	TGCTTTTTAA	GACCCTGAGC	TGTAATCAGA
247_AR_*A.baerii*_consensus	GATCTGGATC	TGGACACTGA	ATTAGGAGTA	TGCTTTTTAA	GACCCTGAGC	TGTAATCAGA
247_AR_*H.huso*_consensus	GATCTGGATC	TGGACACTGA	ATTAGGAGTA	TGCTTTTTAA	GAACCTGAGC	TGTAATCAGA

	65	75	85	95	105	115
	....|....|	....|....|	....|....|	....|....|	....|....|	....|....|
contig_140238_partial_seq	AAAGTAGTTA	TTTACCACAT	GGGTAAAGAT	TTAAGTGTAT	TTG**TAAGGGT**	**CCATGCATGC**
247_AR_*A.ruthenus*_consensus	AAAGTAGTTA	TTTACCACAT	GGGTAAAGAT	TTAAGTGTAT	TTG**TAAGGGT**	**CCATGCATGC**
247_AR_*A.baerii*_consensus	AAAGTAGTTA	TTTACCACAT	GGGTAAAGAT	TTAAGTGTAT	TTG**TAAGGGT**	**CCATGCATGC**
247_AR_*H.huso*_consensus	AAAGTAGTTA	TTTACCACAT	GGGTAAAGAT	TTAAGTGTAT	TTG**TAAGGGT**	**CCATGCATGC**

	125	135	145	155	165	175
	....|....|	....|....|	....|....|	....|....|	....|....|	....|....|
contig_140238_partial_seq	**AG**AAGCATCA	AAGATTATTA	CCTGCCTCCA	TGCTACTGTT	TTACCCTGAT	GAGGTGCGTT
247_AR_*A.ruthenus*_consensus	**AG**AAGCATCA	AAGATTATTA	CCTGCCTCCA	TGCTACTGTT	TTACCCTGAT	GAGGTGCGTT
247_AR_*A.baerii*_consensus	**CT**AAGCATCA	AAGATTATTA	CCTGCCTCCA	TGCTACTGTT	TTACCCTGAT	GAGGTGCGTT
247_AR_*H.huso*_consensus	**CT**AAGCATCA	AAGATTATTA	CCTGCCTCCA	TGCTACTGTT	TTACCCTGAT	GAGGTGCGTT

	185	195	205	215	225	235
	....|....|	....|....|	....|....|	....|....|	....|....|	....|....|
contig_140238_partial_seq	TCAAAAGGAA	AATTCAACTA	GTCACCATGC	TTAAAATGGT	TCGAACATGA	CCCAGATCTC
247_AR_*A.ruthenus*_consensus	TCAAAAGGAA	AATTCAACTA	GTCACCATGC	TTAAAATGGT	TCGAACATGA	CCCAGATCTC
247_AR_*A.baerii*_consensus	TCAAAAGGAA	AATTCAACTA	GTCACGATGC	TTAAAATGGT	TCGAACATGA	CCCAGATCTC
247_AR_*H.huso*_consensus	TCAAAAGGAA	AATTCAACTA	GTCACGATGC	TTAAAATGGT	TCGAACATGA	CCCAGATCTC

```
                                ....|....| ....|....| ....|....| ....|....| ....|....| ....|....|
                                   247        255        265        275        285        295
contig_140238_partial_seq       AAACAGCTGC CATCAATCTA ATTGGGCCAA TGCATTCACA TATAAAAGGA AGGCATTCCT

247_AR_A.ruthenus_consensus     AAACAGCTGC CATCAATCTA ATTGGGCCAA TGCATTCACA TATAAAAGGA AGGCATTCCT

247_AR_A.baerii_consensus       AAACAGCTGC CATCAATCTA ATTGGGCCAA TGCATTCACA TATAAAAGGA AGGCATTCCT

247_AR_H.huso_consensus         AAACAGCTGC CATCAATCTA ATTGGGCCAA TGCATTCACA TATAAAAGGA AGGCATTCCT

                                ....|....| ....|....| ....|....| ....|....| ....|....| ....|....|
                                   305        315        325        335        345        355
contig_140238_partial_seq       ACAGATCTGT AATCCTGTGC AATCTATGCC TTGCCACGGT GCAGCTAAAA TTCAGGGGTG

247_AR_A.ruthenus_consensus     ACAGATCTGT AATCCTGTGC AATCTATGCC TTGCCACGGT GCAGCTAAAA TTCAGGGGTG

247_AR_A.baerii_consensus       ACAGATCTGT AATCCTGTGC AATCTATGCC TTGCCACGGT GCAGCTAAAA TTCAGGGGTG

247_AR_H.huso_consensus         ACAGATCTGT AATCCTGTGC AATCTATGCC TTGCCACGGT GCAGCTAAAA TTCAGAGGTG

                                ....|....| ....|....| ....|....| ....|....| ....|....|
                                   365        375        385        395        405
contig_140238_partial_seq       TTCCTTAAAA ATATAATTAT ACATTAAACA GTAATATACT GCACAATTGT

247_AR_A.ruthenus_consensus     TTCCTTAAAA ATATAATTAT ACATTAAACA GTAATATACT GCACAATTGT

247_AR_A.baerii_consensus       TTCCTTAAAA ATATAATTAT ACATTAAACA GTAATATACT GCACAATTGT

247_AR_H.huso_consensus         TTCCTTAAAA ATATAATTAT ACATTAAACG GTAATATACT GCACAATTGT
```

Figure S1. Alignment of consensus sequence of *H. huso*, *A. ruthenus*, and *A. baerii* to partial sequence of *A. ruthenus* reference contig n. 140238.

Regions in which primers were designed are bold underlined. Diagnostic nucleotides at the 3’ primer-end are grey highlighted.

```
                                   ....|....| ....|....| ....|....| ....|....| ....|....| ....|....|
                                       5          15         25         35         45         55
contig_216845_partial_seq          GATCTGAACA TCAGCCACTA GATAATATTT ATTTTTGTTT GAAGGCTGCA CAGGAACACA

153_HH_A.ruthenus_consensus        GATCTGAACA TCAGCCACTA GATAATATTT ATTTTTGTTT GAAGGCTGCA CAGGAACACA

153_HH_A.baerii_consensus          GATCTGAACA TCAGCCACTA GATAATATTT ATTTTTGTTT GAAGGCTGCA CAGGAACACA

153_HH_H.huso_consensus            GATCTGAACA TCAGCCACTA CATAATATTT ATTTTTGTTT GAAGGCTGCA CAGGAACACA

                                   ....|....| ....|....| ....|....| ....|....| ....|....| ....|....|
                                        65         75         85         95        105        115
contig_216845_partial_seq          TCAGCGTATT GGATCAGTAC TTACTATGTT GCCTTTTGTA GCTTCTCCCA GCAGTCCCCC

153_HH_A.ruthenus_consensus        TCAGCGTATT GGATCAGTAC TTACTATGTT GCCTTTTGTA GCTTCTCCCA ----------

153_HH_A.baerii_consensus          TCAGCGTATT GGATCAGTAC TTACTATGTT GCCTTTTGTA GCTTCTCCCA ----------

153_HH_H.huso_consensus            TCAGCGTATT GGATCAGTAC TTACTATGTT GCCTTTTGTA GCTTCTCCCA ----------

                                   ....|....| ....|....| ....|....| ....|....| ....|....| ....|....|
                                       125        135        145        155        165        175
contig_216845_partial_seq          GAAAGTGATA ACAGGAGACA TACAGGCACA GTAGAGGAAG AGGATGGAGG CCAGGCACTG

153_HH_A.ruthenus_consensus        ---------- ---------- ---------- ---------- ---------- ----------

153_HH_A.baerii_consensus          ---------- ---------- ---------- ---------- ---------- ----------

153_HH_H.huso_consensus            ---------- ---------- ---------- ---------- ---------- ----------
```

Figure S2. Alignment of consensus sequence of *H. huso*, *A. ruthenus*, and *A. baerii* to partial sequence of *A. ruthenus* reference contig n. 216845.

Regions in which primers were designed are bold underlined. Diagnostic nucleotides at the 3’ primer-end are grey highlighted.

Supplementary Note

Characterization of analysed caviar sample

Sample

The caviar purchased in Isetan Store, 60-0022 3-14-1, Shinjuku, Tokyo, Japan.

Species: beluga (*Huso huso*)

Source of caviar: Culture

Country of origin: Iran

Year of re-packing: 2016

Country of re-packing: Germany

Weight: 15g

The label of analysed caviar

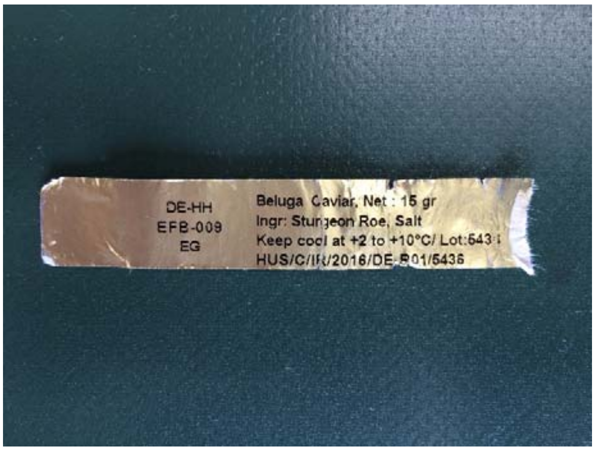

Extraction of DNA

DNA was by NucleoSpin® Tissue Kit (MACHEREY-NAGEL, Germany) using manufacturer protocol with the following modifications:

- One roe was used for extraction. Using more than one roe resulted in poor extraction yield with high contamination that prevented PCR
- The elution volume was 20µl

- The roe initially washed in molecular grade ethanol gave better extraction result and better PCR amplification when compared to fresh roe.

PCR

The PCR mix for tissue samples and roe samples was the same. In cycling program, we increased number of cycles from 30 to 35 for amplification of roe samples.

Results

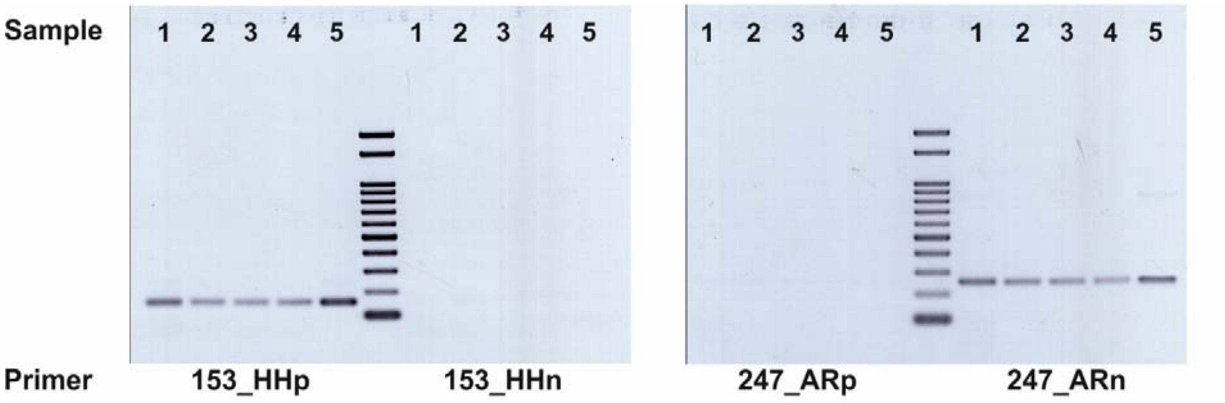

Amplification given by developed primers in the sample of *Huso huso* caviar (1-4) and *H. huso* tissue (5).
